# Tight Bounds Between the Jensen–Shannon Divergence and the Minmax Divergence

**DOI:** 10.3390/e27080854

**Published:** 2025-08-11

**Authors:** Arseniy Akopyan, Herbert Edelsbrunner, Žiga Virk, Hubert Wagner

**Affiliations:** 1 Fora Capital, Miami, FL 33131, USA; 2 ISTA (Institute of Science and Technology Austria), 3400 Klosterneuburg, Austria; 3 Faculty of Computer and Information Science, University of Ljubljana, 1000 Ljubljana, Slovenia; 4 Institute of Mathematics, Physics and Mechanics (IMFM), 1000 Ljubljana, Slovenia; 5 Department of Mathematics, University of Florida, Gainesville, FL 32611, USA

**Keywords:** information theory, relative entropy, Kullback–Leibler divergence, Jensen–Shannon divergence, minmax divergence, Bregman divergence, Burbea–Rao divergence, Jensen-Bregman divergence, metric, bounds

## Abstract

Motivated by questions arising at the intersection of information theory and geometry, we compare two dissimilarity measures between finite categorical distributions. One is the well-known Jensen–Shannon divergence, which is easy to compute and whose square root is a proper metric. The other is what we call the minmax divergence, which is harder to compute. Just like the Jensen–Shannon divergence, it arises naturally from the Kullback–Leibler divergence. The main contribution of this paper is a proof showing that the minmax divergence can be tightly approximated by the Jensen–Shannon divergence. The bounds suggest that the square root of the minmax divergence is a metric, and we prove that this is indeed true in the one-dimensional case. The general case remains open. Finally, we consider analogous questions in the context of another Bregman divergence and the corresponding Burbea–Rao (Jensen–Bregman) divergence.

## 1. Introduction

The starting point of this work is the introduction of the minmax divergence between two finite categorical distributions. It is a special case of a natural measurement arising in certain constructions from computational geometry and topology. Specifically, it coincides with the smallest radius at which two appropriately defined balls intersect. However, since our results promise to be useful also outside the field of geometry and topology, we will tailor the exposition accordingly.

The main result are tight bounds between the minmax divergence and the standard Jensen–Shannon divergence (as well as its generalizations). Given these bounds and the well-known fact the square root of the Jensen–Shannon divergence is a metric, we ask if the same can be proven about the minmax divergence. A supplementary result is a proof that in one dimension its square root is a metric. The general case is left as an open question.

The basic definition of the minmax divergence is based on the classic notion of Shannon entropy and has an intuitive information theoretic interpretation, as will be explained shortly. The definition coincides with an interpretation of the Chernoff information recently provided by Nielsen in [1]. In Section 2, we generalize this definition slightly, allowing us to work with the entire positive orthant of $R^{n}$. Later in Section 5, we further generalize using the language of Bregman divergences, which allows us to work with other notions of entropy, such as the Burg entropy. Before we proceed, we mention that the following exposition is tailored towards this generalization. In particular, we prefer to work with negative Shannon entropy. Being a strictly convex function it allows for a smoother transition to the case of Bregman divergences, which are defined via such functions.

**Shannon entropy and related concepts.** We start from basic definitions and review their information-theoretical interpretations. Consider a random variable that takes one of *n* values. Letting $x=(x_{1},x_{2},\ldots ,x_{n})$ be the vector of probabilities, we can encode the outcome with *expected efficiency* −E(x), in which

(1) $$\begin{array}{cc} E(x) & =\sum_{i=1}^{n}x_{i}\ln x_{i} \end{array}$$

is the (negative) *Shannon entropy* of *x*. We remark that if a binary logarithm was used, this quantity would be expressed in bits. We use natural logarithms to simplify calculations. Suppose we mistakenly assume that the underlying probability vector is $y=(y_{1},y_{2},\ldots ,y_{n})$, and we encode the random variable based on this faulty assumption. The expected efficiency is then −E(y)−〈∇E(y),x−y〉. Comparing this with −E(x), we get

(2) $$\begin{array}{cc} D\left(x∥y\right) & =E(x)-E(y)-\langle \nabla E(y),x-y\rangle \end{array}$$

(3) $$\begin{array}{cc} \; & =\sum_{i=1}^{n}x_{i}\ln\frac{x_{i}}{y_{i}} \end{array}$$

as a measure of the efficiency loss due to the faulty assumption. This quantity is often referred to as the *relative entropy*, which we note is not symmetric. Next assume we know that the random variable is either chosen according to the distribution *x* or according to the distribution *y*, each with 50% likelihood. Our best bet is to encode the result as if the underlying probability distribution were the average, $\mu =\frac{1}{2}\left(x+y\right)$. The expected efficiency is −E(μ), which we compare with $-\frac{1}{2}E\left(x\right)-\frac{1}{2}E\left(y\right)$, the expected efficiency assuming we know from which distribution the random variable is chosen. The difference,

(4) $$\begin{array}{cc} \mathrm{JS}\left(x,y\right) & =\frac{1}{2}E\left(x\right)+\frac{1}{2}E\left(y\right)-E\left(\mu \right) \end{array}$$

(5) $$\begin{array}{cc} \; & =\frac{1}{2}\sum_{i=1}^{n}\left[x_{i}\ln\frac{2x_{i}}{x_{i}+y_{i}}+y_{i}\ln\frac{2y_{i}}{x_{i}+y_{i}}\right] \end{array}$$

is the *Jensen–Shannon divergence*. This can be generalized to non-negative likelihoods ξ+η=1, for which our best bet is to encode using ξx+ηy, with expected divergence ξE(x)+ηE(y)−E(ξx+ηy). There are unique likelihoods, $\xi_{0}+\eta_{0}=1$, for which the expected divergence is maximized. Because *E* is convex, this is also the situation in which the maximum divergence is minimized. We therefore consider this solution our best bet if we do not know how *x* and *y* split the likelihood. Setting $z=\xi_{0}x+\eta_{0}y$, the expected efficiency is −E(z), which we compare with $-\xi_{0}E\left(x\right)-\eta_{0}E\left(y\right)$. The difference,

(6) $$\begin{array}{cc} \mathrm{Mx}\left(x,y\right) & =\xi_{0}E\left(x\right)+\eta_{0}E\left(y\right)-E\left(z\right) \end{array}$$

(7) $$\begin{array}{cc} \; & =\sum_{i=1}^{n}\left[\xi_{0}x_{i}\ln\frac{x_{i}}{z_{i}}+\eta_{0}y_{i}\ln\frac{y_{i}}{z_{i}}\right], \end{array}$$

minimizes the maximum divergence over all possible choices of likelihoods ξ+η=1. We therefore call Mx(x,y) the *minmax divergence* between the two probability distributions. In the next section, we will provide an easier to work with alternative definition.

**Main result.** Our main result is a comparison of the minmax divergence with the standard Jensen–Shannon divergence. Specifically, we prove

(8) $$\begin{array}{c} \frac{e\ln2}{2}\mathrm{Mx}\left(x,y\right)\leq \mathrm{JS}\left(x,y\right)\leq \mathrm{Mx}\left(x,y\right) \end{array}$$

as tight bounds. We remark that the final result uses a generalized form of the above concepts, as defined in the next section.

Given that $\frac{e\ln2}{2}\approx 0.94208$, the two divergences are very close. (As we will argue shortly, it makes sense to consider the square roots of these divergences, in which case the constant is approximately 0.9706, which is even closer to 1.) Additionally, the Jensen–Shannon divergence and the minmax divergence also yield the same intrinsic metric, which is $\frac{1}{4}$ times the intrinsic metric defined by the relative entropy. In the literature, the relative entropy is also known as the *Kullback–Leibler divergence* [2], and the corresponding intrinsic metric is known as the *Fisher information metric* [3]. Both are however not *length metrics*, which means that integrating infinitesimal steps gives a corresponding *intrinsic metric*, which is different from the original metric.

In light of these similarities, we ask if they share more properties. In particular Endres and Schindelin proved that $\sqrt{\mathrm{JS}\left(x,y\right)}$ is a metric [4]. We were able to prove a similar result in dimension one (specifically, in a slightly generalized setting in which $x,y\in R_{+}$). Namely, we prove that $\sqrt{\mathrm{Mx}\left(x,y\right)}$ is a metric. The result in higher dimensions turns out to be challenging, and remains open. We do believe that the geometric proof techniques we introduce are worth sharing, and are likely to be useful in the high-dimensional case (in combination with some other techniques).

**Applications in computational geometry.** We briefly explain how the above concepts can be used in computational geometry and topology and, in particular, why the main result is useful. Our main motivation comes from the field of topological data analysis, a subfield of computational geometry and topology. In short, the idea is to characterize the *shape of data* or, in other words, its geometric-topological structure. We briefly describe a simple case to which our main result is immediately applicable. For a comprehensive treatment of topological data analysis in the context of arbitrary Bregman divergences, which includes our setup with relative entropy, see [5].

In the simplest case—which is also most relevant here—the input data is a finite collection of points. We consider the union of the balls centered at these points and increase their common radius from zero to infinity. As the radius changes, so does the connectivity (or topology) of the union of balls. One basic topological property is the structure of the connected components. At radius zero, each point constitutes its own connected component, and these components may merge as the radius increases. Specifically, two components may merge when two balls develop a nonempty intersection for the first time. (We remark that higher-degree topological features can also be considered, but this requires tools from algebraic topology, which are beyond the scope of this paper.).

This setup can be easily implemented in the Euclidean space. However, in the more interesting situation in which each point is a finite categorical distribution, the balls are better defined using the relative entropy (and not the Euclidean distance), as it allows the outcome to have an information-theoretic interpretation, as outlined above. The radius at which two balls intersect coincides with the minmax divergence [6], whose direct computation (especially for many pairs of points) can be slow. The proposed inequality suggests we compute the approximating Jensen–Shannon divergence instead. It remains fairly accurate while being significantly faster, simpler, and more robust. In particular, this allows for a simple computation of a weighted undirected graph that describes the connected structure of data measured with relative entropy. In topological data analysis language, it would be called the 1-skeleton of the Čech complex (the full complex would be a weighted simplicial complex that encodes intersections between arbitrary tuples of balls and captures higher-degree topological information.).

In summary, our results simplify a fundamental computation in topological data analysis for an important kind of inputs. This is often the first step towards computing a topological descriptor, which has been proven useful in a variety of fields [7] ranging from biology, to astronomy, to materials science. Moreover, if the square root of the minmax divergence is indeed a metric (a fact we were unable to prove beyond dimension one), it would allow one to use existing theoretic in computational tools. Here we mention stability results in topological data analysis that exploit the metric structure of data [8], and classical nearest neighbour search tools that focus on metric spaces [9].

**Outline.** Section 2 introduces the main concepts in their generalized forms and proves some of their fundamental properties. Section 3 shows that the Jensen–Shannon divergence approximates the minmax divergence within a factor 1.061…. Section 4 proves that in one dimension the square root of the minmax divergence is a metric. Section 5 extends the results beyond the Shannon entropy using the framework of Bregman divergences. Section 6 concludes the paper.

**Related work.** Many concepts used in this paper lead back to the seminal work of Claude Shannon [10] on information theory and, in particular, the notion of Shannon entropy. This notion was extended to a dissimilarity measure between two probability distributions by Kullback and Leibler [2], often referred to as the relative (Shannon) entropy or the Kullback–Leibler divergence. The best known metric derived from relative Shannon entropy is based on the Jensen–Shannon divergence, defined by Lin in 1991 [11]. Interestingly, its more general form was introduced a decade earlier by Burbea and Rao [12]. Lew Bregman introduced a notion of Bregman divergence [13], which generalizes the relative entropy. Various other metrics derived from Bregman divergences were studied in [14,15]. More recently, Bregman divergences were further generalized by Nielsen in various ways [16,17,18]. Our work is motivated by results at the intersection of Bregman geometry and computational geometry. The starting point for this direction is the work of Boissonnat, Nielsen and Nock [19,20,21] on computational geometry in the Bregman context.

## 2. Generalized Definitions

In the Introduction, we described basic concepts (such as the relative entropy) along with their information-theoretical interpretation. In this section, we introduce more general versions of these concepts, allowing us to work in the entire positive orthant $R_{+}^{n}$. We remark that the definitions change in subtle ways, and that applying the usual definitions in this extended setup may on occasion be non-sensical.

For the reminder of this paper, we are exclusively concerned with two spaces: the *n*-dimensional *positive orthant*, denoted $R_{+}^{n}$, which consists of all points $x=(x_{1},x_{2},\ldots ,x_{n})$ with $x_{i}>0$ for 1≤i≤n, and the open (n−1)-dimensional *standard simplex*, denoted $\Delta =\Delta^{n-1}$, which consists of the points $x\in R_{+}^{n}$ that satisfy $\sum_{i=1}^{n}x_{i}=1$. A point x∈Δ is really a finite probability distribution, which leads us to believe that Δ is the more important setting. However, $R_{+}^{n}$ is often easier to work with, and we can restrict results to $\Delta \subseteq R_{+}^{n}$.

**Shannon entropy and relative entropy.** Writing lnt for the natural logarithm of t>0, the (negative) *Shannon entropy* is $E:R_{+}^{n}\to R$ defined by $E\left(x\right)=\sum_{i=1}^{n}\left[x_{i}\ln x_{i}-x_{i}\right]$. (Subtracting the extra term is a standard trick to simplify computations while not affecting the interpretation of the resulting relative entropy. However, the interpretation of the Shannon entropy mentioned in the Introduction holds only up to a constant.) We write ${E|}_{\Delta }:\Delta \to R$ for its restriction to the standard simplex.

**Figure 1:**
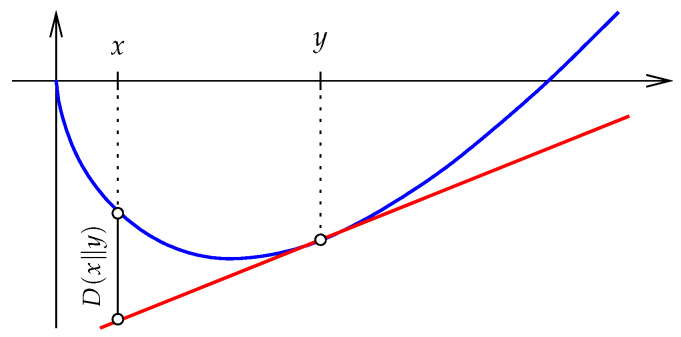
The graph of the Shannon entropy on $R_{+}$, the graph of its best linear approximation at *y*, and the relative entropy from *x* to *y*.

As mentioned in Section 1, −E(x) pertains to the *expected efficiency* of encoding a random variable that distributes according to x∈Δ. If we encode the values assuming the random variable distributes according to y∈Δ, the expected efficiency is the negative of the best linear approximation of *E* at *y* evaluated at *x*, which is −E(y)−〈∇E(y),x−y〉. The relative entropy can be viewed as the non-negative difference between the above approximation and the Shannon entropy of *x*; see Figure 1.

Going back to $x,y\in R_{+}^{n}$, we consider a generalized form of the *relative entropy* from *x* to *y*:

(9) $$\begin{array}{cc} D\left(x∥y\right) & =E(x)-E(y)-\langle \nabla E(y),x-y\rangle \end{array}$$

(10) $$\begin{array}{cc} \; & =\sum_{i=1}^{n}\left[x_{i}\ln\frac{x_{i}}{y_{i}}-x_{i}+y_{i}\right] \end{array}$$

(11) $$\begin{array}{cc} \; & =\sum_{i=1}^{n}D\left(x_{i}∥y_{i}\right). \end{array}$$

Note the extra additive terms, which are absent in the usual form and ensure that the result is nonnegative. When restricted to the standard simplex, it simplifies to its more standard form, namely $\sum_{i=1}^{n}x_{i}\ln\frac{x_{i}}{y_{i}}$.

We say the relative entropy is *decomposable* because it satisfies (11). Observe that the notation separates the points *x* and *y* by a double bar as opposed to a comma to remind us that the measure is generally not symmetric. The relative entropy is also known as the *Kullback divergence*, the *Kullback–Leibler divergence*, or simply the *divergence*; see [3] (page 57). It measures the divergence in encoding efficiency due to assuming a different distribution.

**Figure 2:**
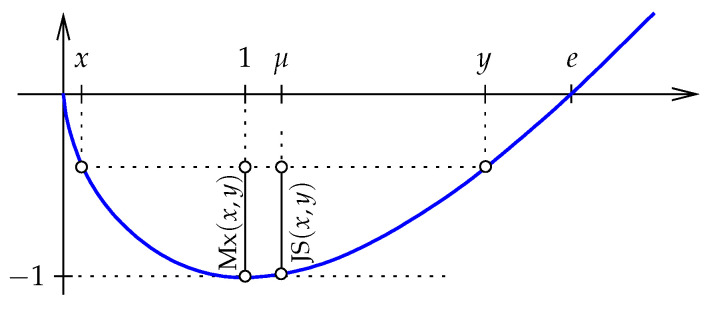
A pair $x,y\in R_{+}$ that satisfies E(x)=E(y), the midpoint, μ=(x+y)/2, and the minmax divergence center z=1. The Jensen–Shannon divergence and the minmax divergence of *x* and *y* are labeled.

**Jensen–Shannon divergence.** Similar to the relative entropy, the *Jensen–Shannon divergence* generalized to the positive orthant is a function $\mathrm{JS}:R_{+}^{n}\times R_{+}^{n}\to R$, but it is symmetric by taking the average relative entropy from *x* to $\mu =\frac{1}{2}\left(x+y\right)$ and from *y* to μ; see Figure 2:

(12) $$\begin{array}{cc} \mathrm{JS}\left(x,y\right) & =\frac{1}{2}\left[D\left(x∥\mu \right)+D\left(y∥\mu \right)\right] \end{array}$$

(13) $$\begin{array}{cc} \; & =\frac{1}{2}E\left(x\right)+\frac{1}{2}E\left(y\right)-E\left(\mu \right) \end{array}$$

(14) $$\begin{array}{cc} \; & =\frac{1}{2}\sum_{i=1}^{n}\left[x_{i}\ln\frac{2x_{i}}{x_{i}+y_{i}}+y_{i}\ln\frac{2y_{i}}{x_{i}+y_{i}}\right]. \end{array}$$

(15) $$\begin{array}{cc} \; & =\sum_{i=1}^{n}\mathrm{JS}\left(x_{i},y_{i}\right), \end{array}$$

in which we get (13) by noting (x−μ)+(y−μ)=0. Similar to the relative entropy, the Jensen–Shannon divergence is decomposable (15). As pointed out in [4], JS(x,y) measures the divergence of expected efficiency when we encode a random variable that distributes half of the time according to x∈Δ and the other half of the time according to y∈Δ using the average of *x* and *y*.

It is not difficult to prove that if we substitute any other point for μ, then the average relative entropy and therefore the divergence in efficiency increases. For reasons that will become obvious shortly, we prove this optimality result for a weighted version of this divergence. Let ξ+η=1 and set w=ξx+ηy. The corresponding *weighted Jensen–Shannon divergence* is

(16) $$\begin{array}{cc} {\mathrm{JS}}_{\xi }\left(x∥y\right) & =\xi D\left(x∥w\right)+\eta D\left(y∥w\right) \end{array}$$

(17) $$\begin{array}{cc} \; & =\xi E(x)+\eta E(y)-E(w) \end{array}$$

(18) $$\begin{array}{cc} \; & =\sum_{i=1}^{n}\left[\xi x_{i}\ln\frac{x_{i}}{w_{i}}+\eta y_{i}\ln\frac{y_{i}}{w_{i}}\right] \end{array}$$

(19) $$\begin{array}{cc} \; & =\sum_{i=1}^{n}{\mathrm{JS}}_{\xi }\left(x_{i}∥y_{i}\right). \end{array}$$

To prove optimality, we set $f_{\xi }\left(u\right)=\xi D\left(x∥u\right)+\eta D\left(y∥u\right)$, noting that $f_{\xi }\left(w\right)={\mathrm{JS}}_{\xi }\left(x∥y\right)$. The following lemma and proof can also be found in [22].

**Lemma 1** 
(Optimality of Weighted JS)**.** *Let $x,y\in R_{+}^{n}$, ξ+η=1, and w=ξx+ηy. Then $f_{\xi }\left(w\right)\leq f_{\xi }\left(u\right)$ for every $u\in R_{+}^{n}$, with equality iff u=w.*

**Proof.**  Computing the difference, $X=f_{\xi }\left(u\right)-f_{\xi }\left(w\right)$, most terms cancel and we get

(20) $$\begin{array}{cc} X & =\xi \left[D\left(x∥u\right)\;-\;D\left(x∥w\right)\right]+\eta \left[D\left(y∥u\right)\;-\;D\left(y∥w\right)\right] \end{array}$$

(21) $$\begin{array}{cc} \; & =E(w)-E(u)-\langle \nabla E(u),w-u\rangle , \end{array}$$

in which we use ξ(x−w)+η(y−w)=0. In other words, the difference is equal to D(w∥u), which is non-negative and zero iff u=w by the strict convexity of *E*. □

**Remark 1.** 
*To get an information theoretic interpretation of the result, we assume x,y∈Δ and suppose a random variable that distributes according to x with likelihood 0≤ξ≤1 and according to y with likelihood η=1−ξ. Lemma 1 says that our best bet is to encode with w=ξx+ηy. In words, w minimizes the weighted Jensen–Shannon divergence.*

**Minmax divergence.** Similar to the Jensen–Shannon divergence, the *minmax divergence* is a symmetric function $\mathrm{Mx}:R_{+}^{n}\times R_{+}^{n}\to R$. It is defined by mapping x,y to the larger relative entropy to a third point, $z\in R_{+}^{n}$, in which *z* is selected so as to minimize this maximum:

(22) $$\begin{array}{cc} \mathrm{Mx}\left(x,y\right) & =\underset{z\in R_{+}^{n}}{\inf}\max\left\{D\left(x∥z\right),D\left(y∥z\right)\right\}. \end{array}$$

We call the point *z* that gives the infimum the *minmax divergence center* of the pair. We remark that the general form of minmax divergence introduced in Section 5 generalizes the Chernoff information [1] popular in statistics.

As proved for example in [6], *z* is a convex combination of *x* and *y*. We strengthen this result by proving that it is the particular convex combination that maximizes the weighted Jensen–Shannon divergence, and that this weighted Jensen–Shannon divergence equals the minmax divergence.

**Lemma 2**  (Minmax-Maxweight)**.** *Let $x,y\in R_{+}^{n}$ and $\xi_{0}+\eta_{0}=1$ such that $z_{0}=\xi_{0}x+\eta_{0}y$ is the minmax divergence center. Then $\mathrm{Mx}\left(x,y\right)={\mathrm{JS}}_{\xi_{0}}\left(x∥y\right)\geq {\mathrm{JS}}_{\xi }\left(x∥y\right)$ for all ξ.*

**Proof.**  Let ξ+η=1 and write z=ξx+ηy for a general affine combination of *x* and *y*. The restriction of *E* to the line of such points is a strictly convex function. It follows that there is a unique affine combination, $z_{0}=\xi_{0}x+\eta_{0}y$, such that $D\left(x∥z_{0}\right)=D\left(y∥z_{0}\right)$. The weighted Jensen–Shannon divergence at $z_{0}$ is ${\mathrm{JS}}_{\xi_{0}}\left(x∥y\right)=\xi_{0}D\left(x∥z_{0}\right)+\eta_{0}D\left(y∥z_{0}\right)$, which is equal to $D\left(x∥z_{0}\right)=D\left(y∥z_{0}\right)$, as claimed.

By convexity of the restriction of *E*, the maximum relative entropy is larger than this shared value for every affine combination $z\neq z_{0}$. Similarly, the weighted Jensen–Shannon divergence is smaller than ${\mathrm{JS}}_{\xi_{0}}\left(x∥y\right)$ at every affine combination $z\neq z_{0}$. □

**Remark 2.** 
*In contrast to the other measures discussed so far, the minmax divergence is not decomposable.*

**Remark 3.** 
*Since the minmax divergence center of x and y lies between these two points, it is constrained to a compact set so we can replace the infimum in *(22)* by a minimum. This justifies the name of corresponding divergence. Lemma 2 says that the minimum of the maximum relative entropy is equal to the maximum weighted Jensen–Shannon divergence, which justifies the name of the lemma.*

**Explicit formula.** While the minmax divergence is defined as an infimum, it is possible to compute it explicitly. To state the formula, we introduce $G:R_{+}\to R$ defined by

(23) $$\begin{array}{cc} G(t) & =\frac{t^{\frac{t}{t-1}}}{e}-\frac{t\ln t}{t-1}. \end{array}$$

It is well defined at all positive t≠1, and we get G(1)=0 in the limit because $\lim_{t\to 1}t^{\frac{t}{t-1}}=e$ and $\lim_{t\to 1}\frac{t\ln t}{t-1}=1$ by the rule de l’Hôpital.

**Lemma 3** 
(Minmax divergence Formula)**.** *For $x,y\in R_{+}$, we have $\mathrm{Mx}\left(x,y\right)=xG(\frac{y}{x})$.*

**Proof.**  For x=y both sides vanish, so the relation holds. We therefore assume x≠y for the remainder of the proof. Letting *z* be the minmax divergence center of *x* and *y*, we recall that the derivative at *z* is the slope of the line that passes through (x,E(x)) and (y,E(y)): $E^{\prime }\left(z\right)=\ln z=\left[E\left(y\right)-E\left(x\right)\right]/\left[y-x\right]$. We express this equation in terms of $\frac{y}{x}$:

(24) $$\begin{array}{cc} \ln z & =\frac{y\ln y-x\ln x-(y-x)}{y-x} \end{array}$$

(25) $$\begin{array}{cc} \; & =\frac{\frac{y}{x}\left(\ln\frac{y}{x}+\ln x\right)-\ln x}{\frac{y}{x}-1}-1 \end{array}$$

(26) $$\begin{array}{cc} \; & =\frac{\frac{y}{x}\ln\frac{y}{x}}{\frac{y}{x}-1}+\ln x-1. \end{array}$$

Write $A=\left(\frac{y}{x}\ln\frac{y}{x}\right)/\left(\frac{y}{x}-1\right)$ for the first term on the right-hand side of (26). Recall that the minmax divergence of *x* and *y* is the vertical distance between the point (z,E(z)) and the line that passes through (x,E(x)) and (y,E(y)). By construction, the slope of this line is lnz. We express the vertical distance as the sum of two vertical distances, which we then express in terms of *A* and $\frac{y}{x}$:

(27) $$\begin{array}{cc} \mathrm{Mx}\left(x,y\right) & =[(z-x)\ln z]+[E(x)-E(z)] \end{array}$$

(28) $$\begin{array}{cc} \; & =z-x+x\ln x-x\ln z \end{array}$$

(29) $$\begin{array}{cc} \; & =z-x+x\ln x-x[A+\ln x-1] \end{array}$$

(30) $$\begin{array}{cc} \; & =x[e^{A-1}-A] \end{array}$$

(31) $$\begin{array}{cc} \; & =xG\left(\frac{y}{x}\right), \end{array}$$

in which we use (26) to get (29), we cancel −x+xlnx and replace *z* to get (30), and finally use $\frac{y}{x}=e^{\ln\frac{y}{x}}$ to get (31). □

**Bregman divergences.** The relative entropy can be viewed from the perspective of Bregman divergences [13]. Indeed, it is an instance of a Bregman divergence generated by the negative Shannon entropy. We briefly introduce the setup for general Bregman divergences, generated by arbitrary convex functions, or more technically functions of Legendre type.

Given an open convex set $\Omega \subseteq R^{d}$, a function F:Ω→R is of Legendre type if it is (1) differentiable, (2) strictly convex and (3) the magnitude of its gradient diverges to positive infinity when evaluated at points converging to the boundary of the domain. Given a function *F* of Legendre type, the Bregman divergence [13] generated by *F* is defined as

$$\begin{array}{c} D_{F}:\Omega \times \Omega \to R,\;D_{F}\left(x∥y\right)=F\left(x\right)-\left(F\left(y\right)+\langle \nabla F\left(y\right),x-y\rangle \right). \end{array}$$

We will use this concept to generalize our main result in Section 5.

## 3. Comparison of Divergences

We think of the Jensen–Shannon divergence as a readily computed approximation of the minmax divergence. The approximation is very close, and we prove in this section that the Jensen–Shannon divergence is always between $\frac{e\ln2}{2}=0.942\ldots$ and 1 times the minmax divergence. We prove this first in one dimension and then generalize the result to *n* dimensions.

**Approximation with ellipse.** The main tool in proving the relation between the minmax divergence and the Jensen–Shannon divergence is the—surprisingly close—approximation of the graph of the Shannon entropy defined over $R_{+}$ with an arc of an ellipse. We are interested in the interval [0,e], so we choose the ellipse to

- pass through the points (0,0) and (e,0);
- have its minimum at the point (1,−1);
- have the same curvature at (1,−1) as the graph of the Shannon entropy.

Writing the ellipse as the zero-set of a function, we introduce $\Gamma :R^{2}\to R$ defined by

(32) $$\begin{array}{cc} \Gamma (x_{1},x_{2}) & =x_{1}^{2}+\left(2-e\right)x_{1}x_{2}+\left(3-e\right)x_{2}^{2}\\ \; & \;\;\;\;-ex_{1}+\left(2-e\right)x_{2}. \end{array}$$

**Figure 3:**
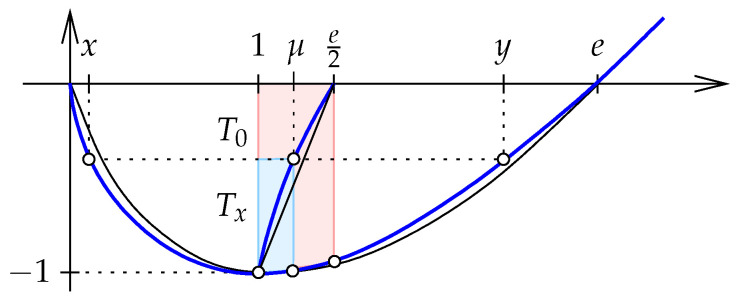
The (blue) midpoint line of *E* lies to the left of the (black) midpoint line of *G*. The two shaded trapezoids visualize the ratios between the minmax divergence and the Jensen–Shannon divergence for the pairs 0<e and 0<x<y<e with E(x)=E(y). Since the upper right corner of the smaller trapezoid lies on the midpoint line of *E*, the second ratio is smaller than the first ratio.

It is not difficult to check that $\Gamma^{-1}\left(0\right)$ satisfies the above three properties. We also note that $\Gamma (x_{1},x_{2})$ is negative for points inside the ellipse and positive for points outside the ellipse. Within [0,e], the approximation of *E* by the lower portion of the ellipse is astonishingly close, and we exaggerate the difference in Figure 3 to make it visible. We prove that from 0 to 1 the graph of *E* is below the ellipse, and from 1 to *e* it is above the ellipse.

**Lemma 4**  (Below-Above)**.** *Letting $E:R_{+}\to R$ be the 1-dimensional Shannon entropy and $\Gamma :R^{2}\to R$ the function defined in *(32)*. Then*

(33) $$\begin{array}{c} \Gamma \left(x,E\left(x\right)\right)\left\{\begin{array}{cc} >0 & \mathrm{for}\;0<x<1,\\ <0 & \mathrm{for}\;1<x<e. \end{array}\right. \end{array}$$

**Proof.**  Write Γ(x,E(x))=xf(x), in which

(34) $$\begin{array}{cc} f(x) & =(2x-2)+(2-e+ex-4x)\ln x\\ \; & \;\;\;\;+\left(3-e\right)x\ln^{2}x. \end{array}$$

We have f(1)=f(e)=0 by construction of Γ, but not necessarily f(0)=0 because we divided by *x*. It suffices to prove that f(x) is positive for 0<x<1 and negative for 1<x<e. Next we compute the first two derivatives, again after removing a monotonic factor to simplify the computations. Specifically, we write $f^{\prime }\left(x\right)=\frac{1}{x}g\left(x\right)$, in which

(35) $$\begin{array}{cc} g(x) & =\left(e-2\right)\left(x-1-x\ln x\right)+\left(3-e\right)x\ln^{2}x, \end{array}$$

(36) $$\begin{array}{cc} g^{\prime }\left(x\right) & =\left(3-e\right)\ln^{2}x-\left(3e-8\right)\ln x. \end{array}$$

The derivative of *g* is quadratic in lnx, with zeros at x=1 and $x=u_{0}=\exp\left(\frac{3e-8}{3-e}\right)=1.732\ldots$. Hence, $g^{\prime }\left(x\right)$ is negative for $1<x<u_{0}$ and positive outside the corresponding closed interval. Returning to *g*, we note that *g* is zero, negative, positive at $1<u_{0}<e$:

(37) $$\begin{array}{cc} g(1) & =0,\;g\left(u_{0}\right)=-0.010\ldots ,\;g\left(e\right)=0.047\ldots . \end{array}$$

Our analysis of $g^{\prime }$ implies that *g* increases from 0 to 1, it decreases from 1 to $u_{0}$, and finally it increases again from $u_{0}$ to *e*, with a zero at some value $u_{1}$ between $u_{0}$ and *e*. Hence, *f* decreases from 0 to $u_{1}$, with $1<u_{1}<e$, and it increases from $u_{1}$ to *e*. Since f(1)=f(e)=0, this implies that *f* is positive from 0 to 1 and negative from 1 to *e*. The claimed inequalities for Γ follow. □

**Midpoint lines.** We are interested in the relative position of two midpoint lines. The first is defined by the Shannon entropy, *E*, and the second by the function G:[0,e]→R whose graph is the portion of the ellipse on and below the horizontal coordinate axis. Both lines consist of points $\left(\frac{1}{2}\left(x+y\right),t\right)$ with 0≤x≤1≤y≤e, in which the points of the first line satisfy E(x)=E(y)=t and the points of the second line satisfy G(x)=G(y)=t.

The ellipse can be obtained by shearing a circle, and since this operation takes straight lines to straight lines, it follows that the midpoint line of *G* is a straight line segment. Its endpoints are (1,−1) and $\left(\frac{e}{2},0\right)$. By Lemma 4, the midpoint line of *E* is a curve that connects the same two endpoints but lies otherwise to the left of the line segment; see Figure 3.

**Inequalities.** Recall the definition of the weighted Jensen–Shannon divergence for a real parameter and points:

(38) $$\begin{array}{cc} {\mathrm{JS}}_{\xi }\left(x∥y\right) & =\xi E(x)+\eta E(y)-E(z), \end{array}$$

in which η=1−ξ and z=ξx+ηy. The (unweighted) Jensen–Shannon divergence is $\mathrm{JS}\left(x,y\right)={\mathrm{JS}}_{1/2}\left(x∥y\right)$, and from Lemma 2 we know that the minmax divergence can be written as $\mathrm{Mx}\left(x,y\right)=\max_{\xi }{\mathrm{JS}}_{\xi }\left(x∥y\right)$. We prove that the two measures of information divergence are good approximations of each other.

**Theorem 1** 
(Loss Comparison)**.** *Letting $x,y\in R_{+}^{n}$, we have $\frac{e\ln2}{2}\;\mathrm{Mx}\left(x,y\right)\leq \mathrm{JS}\left(x,y\right)\leq \mathrm{Mx}\left(x,y\right)$.*

**Proof.**  The upper bound on JS(x,y) follows from Lemma 2, so we focus on proving the lower bound. We begin with the 1-dimensional case, when n=1. Recall that D(Cx∥Cy)=CD(x∥y) for every C≥0. Hence,

(39) $$\begin{array}{cc} \mathrm{JS}\left(Cx,Cy\right) & =C\;\mathrm{JS}\left(x,y\right), \end{array}$$

(40) $$\begin{array}{cc} \mathrm{Mx}\left(Cx,Cy\right) & =C\;\mathrm{Mx}\left(x,y\right), \end{array}$$

which implies that the ratio is independent of *C*. Given x<y, we can find C>0 such that E(Cx)=E(Cy), so we assume E(x)=E(y) for the remainder of the 1-dimensional argument. This implies that the minmax divergence center is z=1. Setting x=0 and y=e, we have E(0)=E(e)=0, and the ratio is

(41) $$\begin{array}{cc} \frac{\mathrm{Mx}\left(0,e\right)}{\mathrm{JS}\left(0,e\right)} & =\frac{-1}{E(\frac{e}{2})}=\frac{-1}{\frac{e}{2}\ln\frac{e}{2}-\frac{e}{2}}=\frac{2}{e\ln2}. \end{array}$$

Observe that this ratio is the length of the left edge divided by the length of the right edge of the trapezoid $T_{0}$ in Figure 3. For a general pair 0<x<y with E(x)=E(y), we represent the ratio by the left and right edges of a similar trapezoid, $T_{x}$. Importantly, the two trapezoids share (1,−1) as their common lower left corner, and the bottom edge of $T_{x}$ has smaller positive slope than the bottom edge of $T_{0}$. The height of $T_{0}$ is 1 and that of $T_{x}$ is t<1. To compare the two ratios, we thus consider $T_{x}/t+\left(1-t,t-1\right)$, which is the scaled version of $T_{x}$ whose lower left corner is (1,−1) and whose height is 1. The upper right corner of $T_{x}$ lies on the midpoint line of *E*, which by Lemma 4 implies that the upper right corner of the scaled trapezoid lies to the left of $\frac{e}{2}$ on the horizontal coordinate axis. It follows that the width of the scaled trapezoid is smaller than the width of $T_{0}$. Hence,

(42) $$\begin{array}{c} \frac{\mathrm{Mx}\left(x,y\right)}{\mathrm{JS}\left(x,y\right)}<\frac{\mathrm{Mx}\left(0,e\right)}{\mathrm{JS}\left(0,e\right)}=\frac{2}{e\ln2}. \end{array}$$

We get equality for x=0 and for x=1, which implies the claimed lower bound for n=1 dimension. Moving on to n≥1 dimensions, we recall that the minmax divergence center is a convex combination of the two points [6]. Specifically, the center satisfies z=ξx+ηy with ξ,η≥0 and ξ+η=1. Using Lemma 2, the decomposability of the weighted Jensen–Shannon divergence (19), and the claimed inequality in n=1 dimension—in this sequence—we get

(43) $$\begin{array}{cc} \mathrm{Mx}\left(x,y\right) & ={\mathrm{JS}}_{\xi }\left(x∥y\right) \end{array}$$

(44) $$\begin{array}{cc} \; & =\sum_{i=1}^{n}{\mathrm{JS}}_{\xi }\left(x_{i}∥y_{i}\right) \end{array}$$

(45) $$\begin{array}{cc} \; & \leq \frac{2}{e\ln2}\sum_{i=1}^{n}\mathrm{JS}\left(x_{i},y_{i}\right) \end{array}$$

(46) $$\begin{array}{cc} \; & =\frac{2}{e\ln2}\mathrm{JS}\left(x,y\right), \end{array}$$

as claimed. □

**Remark 4.** 
*The bounds in Theorem 1 are tight. To see this for the lower bound, we note that Mx(0,e)=1 and $\mathrm{JS}\left(0,e\right)=-E\left(\frac{e}{2}\right)=\frac{e\ln2}{2}$. While 0 is formally not part of the domain, we can take points arbitrarily close to 0 and thus get the bound in the limit. To see that the upper bound is tight, we let ε>0 be small and set x=1−ε and y=1+ε. We can see the two entropies geometrically as the vertical distance of two points on the graph of E below the line that passes through (x,E(x)) and (y,E(y)); see Figure 2. For JS(x,y) this point is (μ,E(μ))=(1,−1), and for Mx(x,y) this point is (z,E(z)), in which z is the minmax divergence center of x and y. To determine z, we note that D(x∥z)=D(y∥z). After some computations, including the Taylor expansions of lns around s=1 and of $e^{s}$ around s=0, we find that z is $1-\frac{1}{3}\varepsilon^{2}$ plus a fourth-order term in ε. In words, z approaches μ=1 much faster than a and b. It follows that in the limit, the two entropies are the same, as required.*

## 4. Proof of Metric in Dimension One

As proved in [4], the square root of the Jensen–Shannon divergence is a metric in $R_{+}$. Using the decomposability of the Jensen–Shannon divergence together with the Minkowski inequality, it is then easy to prove that this square root is also a metric in $R_{+}^{n}$. We prove that the square root of the minmax divergence is a metric in $R_{+}$. Since the minmax divergence is not decomposable, we do not know yet whether its square root is a metric in $R_{+}^{n}$.

**The ratio method.** Suppose A:R×R→R satisfies the triangle inequality and B:R×R→R is another function on the product. To prove that *B* also satisfies the triangle inequality, we may consider the ratio, f(x,y)=B(x,y)/A(x,y) and prove its *monotonicity*, that is:

(47) $$\begin{array}{cc} f(a,b) & \leq f(x,y) \end{array}$$

whenever a≤x≤y≤b.

**Lemma 5** 
(Triangle Inequality)**.** *Let A,B,f:R×R→R in which A satisfies the triangle inequality and f=B/A is monotonic. Then B satisfies the triangle inequality.*

**Proof.**  Let x≤y≤z. Then

(48) $$\begin{array}{cc} \;\;\;\;B(x,y)\;+\;B(y,z) & =f(x,y)A(x,y)\;+\;f(y,z)A(y,z) \end{array}$$

(49) $$\begin{array}{cc} \; & \geq f(x,z)A(x,z) \end{array}$$

(50) $$\begin{array}{cc} \; & =B(x,z), \end{array}$$

in which we get (49) using the monotonicity of *f* and the triangle inequality for *A*. □

To apply the lemma, we set $B\left(x,y\right)=\sqrt{\mathrm{Mx}\left(x,y\right)}$ and $A\left(x,y\right)=|\sqrt{y}-\sqrt{x}|$. Clearly, *A* is a metric in $R_{+}$, so it will suffice to show that the ratio is monotonic.

**A first application.** As a warm up exercise, we use the ratio method expressed in Lemma 5 to re-prove the main result of [4].

**Theorem 2** 
(JS Revisited)**.** *Let $E:R_{+}^{n}\to R$ be the Shannon entropy and $\mathrm{JS}:R_{+}^{n}\times R_{+}^{n}\to R$ map every pair to the Jensen–Shannon divergence. Then $\sqrt{\mathrm{JS}}$ is a metric in $R_{+}^{n}$.*

**Proof.**  We begin with the one-dimensional case, n=1. It is clear that JS(x,y) is non-negative, zero iff x=y, and symmetric. It remains to prove that its square root satisfies the triangle inequality. Setting

(51) $$\begin{array}{cc} f(x,y) & =\frac{\sqrt{\mathrm{JS}\left(x,y\right)}}{|\sqrt{y}-\sqrt{x}|}, \end{array}$$

we will prove that *f* is monotonic as defined in (47). Recall that JS(Cx,Cy)=CJS(x,y) for every C>0. It follows that f(Cx,Cy)=f(x,y), which allows us to assume x=1. To simplify the notation, we set $t^{2}=y$ assuming $t^{2}\geq 1$. Writing $g\left(t\right)=\mathrm{JS}\left(1,t^{2}\right)/{\left(t-1\right)}^{2}=f{\left(1,y\right)}^{2}$, we aim at proving that *g* is monotonically decreasing, that is: $g^{\prime }\left(t\right)<0$ for all t≥1. Recall from (5) that

(52) $$\begin{array}{cc} \mathrm{JS}\left(1,t^{2}\right) & =\frac{1}{2}\left[\ln\frac{2}{1+t^{2}}+t^{2}\ln\frac{2t^{2}}{1+t^{2}}\right], \end{array}$$

(53) $$\begin{array}{cc} \frac{\partial \mathrm{JS}\left(1,t^{2}\right)}{\partial t} & =t\ln\frac{2t^{2}}{t^{2}+1}. \end{array}$$

The derivative of *g* is $g^{\prime }\left(t\right)=N\left(t\right)/{\left(t-1\right)}^{3}$, in which

(54) $$\begin{array}{cc} N(t) & =\left(t-1\right)\frac{\partial \mathrm{JS}\left(1,t^{2}\right)}{\partial t}-2\mathrm{JS}\left(1,t^{2}\right) \end{array}$$

(55) $$\begin{array}{cc} \; & =-t\ln t^{2}+\left(t^{2}+1\right)\ln\frac{t^{2}+1}{2}. \end{array}$$

For t=1, both the numerator and the denominator vanish: N(1)=0 and D(1)=0 in which $D\left(t\right)={\left(t-1\right)}^{3}$. Applying the rule de l’Hôpital three times, we get

(56) $$\begin{array}{cc} N^{\prime }\left(t\right) & =\ln\frac{t^{2}+1}{2}-\ln t^{2}+\frac{2t-2}{t^{2}+1}, \end{array}$$

(57) $$\begin{array}{cc} N^{\prime \prime }\left(t\right) & =-\frac{2\left(t+1\right){\left(t-1\right)}^{2}}{{\left(t^{2}+1\right)}^{2}t}, \end{array}$$

(58) $$\begin{array}{cc} N^{\prime \prime \prime }\left(t\right) & =\frac{4t^{7}-6t^{6}-8t^{5}+6t^{4}-12t^{3}+14t^{2}+1}{{\left(t^{5}+2t^{3}+t\right)}^{2}}, \end{array}$$

with $N^{\prime }\left(1\right)=N^{\prime \prime }\left(1\right)=N^{\prime \prime }\left(1\right)=0$. However, $D^{\prime }\left(1\right)=D^{\prime \prime }\left(1\right)=0$ and $D^{\prime \prime \prime }\left(1\right)=6$. Hence, $g^{\prime }\left(1\right)=0$ and it suffices to prove $g^{\prime \prime }\left(t\right)<0$ for t>1. Since the denominator of $g^{\prime }$ is positive, this is equivalent to $N^{\prime }\left(t\right)<0$ for t>1. But this follows from $N^{\prime }\left(1\right)=0$ and $N^{\prime \prime }\left(t\right)<0$ for t>1, which can be seen from (57). Hence *f* is monotonic and since the denominator in (51) satisfies the triangle inequality, Lemma 5 implies that $\sqrt{\mathrm{JS}}$ also satisfies the triangle inequality and therefore is a metric.

Having established the claim in one dimension, we get the *n*-dimensional result using the Minkowski inequality, which for non-negative real numbers $a_{i}$ and $b_{i}$ implies

(59) $$\begin{array}{cc} \sqrt{\sum_{i=1}^{n}{\left(a_{i}+b_{i}\right)}^{2}} & \leq \sqrt{\sum_{i=1}^{n}a_{i}^{2}}+\sqrt{\sum_{i=1}^{n}b_{i}^{2}}. \end{array}$$

Recall that the Jensen–Shannon divergence is *decomposable*: $\mathrm{JS}\left(x,y\right)=\sum_{i=1}^{n}\mathrm{JS}\left(x_{i},y_{i}\right)$ for $x,y\in R_{+}^{n}$. Letting $z\in R_{+}^{n}$ be a third point, we set $a_{i}^{2}=\mathrm{JS}\left(x_{i},y_{i}\right)$ and $b_{i}^{2}=\mathrm{JS}\left(y_{i},z_{i}\right)$ for all *i*. Since $\sqrt{\mathrm{JS}}$ satisfies the triangle inequality in one dimension, we have ${\left(a_{i}+b_{i}\right)}^{2}\geq \mathrm{JS}\left(x_{i},z_{i}\right)$ for 1≤i≤n. It follows that the left-hand side of (59) is larger than or equal to $\sqrt{\mathrm{JS}\left(x,z\right)}$. The right-hand side of (59) is equal to $\sqrt{\mathrm{JS}\left(x,y\right)}+\sqrt{\mathrm{JS}\left(y,z\right)}$, which implies the triangle inequality for the square root of the Jensen–Shannon divergence in *n* dimensions. □

**Figure 4:**
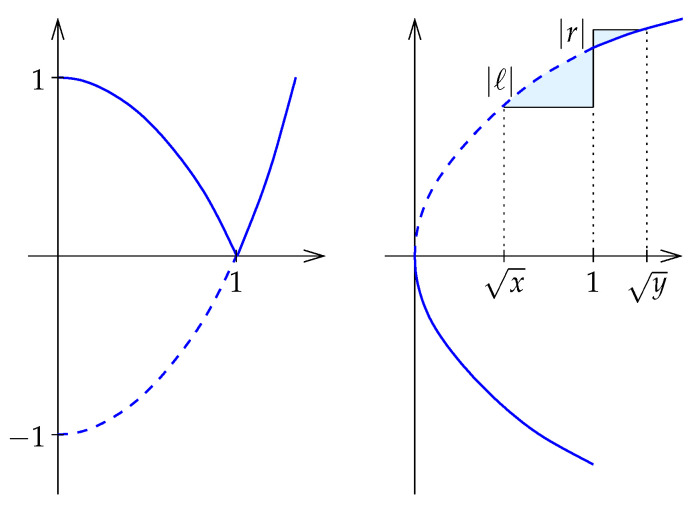
**Left**: the (solid) graph of *F* and the (partially dotted) graph of $\bar{F}$. **Right**: the (solid) graph of $F^{\prime }$ and the (partially dotted) graph of ${\bar{F}}^{\prime }$. The shaded regions have area |ℓ| and |r|, as discussed in the proof of Theorem 3.

**Further preparations.** Recall that the Shannon entropy is defined by E(t)=tlnt−t. The related function, $F:R_{+}\to R$, defined by

(60) $$\begin{array}{cc} F(t) & =\sqrt{E(t^{2})+1} \end{array}$$

(61) $$\begin{array}{cc} \; & =\sqrt{2t^{2}\ln t-t^{2}+1}, \end{array}$$

will play a crucial role in the proof of our next theorem; see Figure 4. The derivative has a discontinuity at t=1, but if we reflect the preceding branch to get $\bar{F}:R_{+}\to R$ defined by $\bar{F}\left(t\right)=-F\left(t\right)$ for 0<t≤1 and $\bar{F}\left(t\right)=F\left(t\right)$ for 1≤t, we obtain a convex function; see again Figure 4 on the left. Appendix A proves that $\bar{F}$ is convex and everywhere differentiable, and that its derivative, ${\bar{F}}^{\prime }$, is concave; see again Figure 4.

**One dimension.** We are now ready to prove that the square root of the minmax divergence is a metric in one dimension.

**Theorem 3** 
(1D Metric)**.** *Let $E:R_{+}\to R$ be the Shannon entropy and $\mathrm{Mx}:R_{+}\times R_{+}\to R$ map every pair to its minmax divergence. Then $\sqrt{\mathrm{Mx}}$ is a metric in $R_{+}$.*

**Proof.**  It is clear that $\sqrt{\mathrm{Mx}\left(x,y\right)}$ is non-negative, zero iff x=y, and symmetric. It thus remains to prove that it satisfies the triangle inequality. Setting

(62) $$\begin{array}{cc} f(x,y) & =\frac{\sqrt{\mathrm{Mx}\left(x,y\right)}}{|\sqrt{y}-\sqrt{x}|}, \end{array}$$

we will prove shortly that *f* is monotonic as defined in (47). Lemma 5 then implies that $\sqrt{\mathrm{Mx}}$ satisfies the triangle inequality. It thus remains to prove f(a,b)≤f(x,y) whenever a≤x≤y≤b. We begin by noting that we may assume these intervals are *canonical*, by which we mean that E(a)=E(b) and E(x)=E(y). Indeed, if E(x)≠E(y), then we can find C>0 such that E(Cx)=E(Cy), which then implies f(Cx,Cy)=f(x,y).

**Figure 5:**
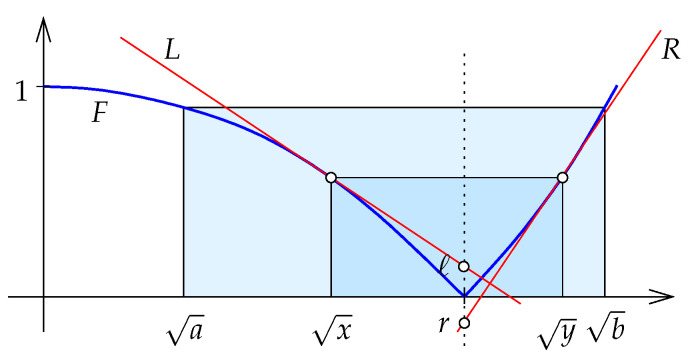
To improve visibility, we draw the graph of *F* stretched in the horizontal direction. The aspect ratios of the shaded rectangles are the values of *f* at x,y and at a,b. The tangent lines *L* at $t=\sqrt{x}$ and *R* at $t=\sqrt{y}$ intersect at a point above the zero line.

We now use the function $f:R_{+}\to R$ defined by $F\left(t\right)=\sqrt{E(t^{2})+1}$ to draw a geometric picture of the situation; see Figure 5.

To prove monotonicity, that is: f(a,b)≤f(x,y), we consider the linear functions L,R:R→R that satisfy L(t)=F(t), for $t=\sqrt{a},\sqrt{x}$, and R(t)=F(t), for $t=\sqrt{y},\sqrt{b}$. The goal is to show that the aspect ratio of the rectangle defined by a,b is less than that defined by x,y. Equivalently, we show that the point at which the lines *L* and *R* meet has positive second coordinate. By the differentiability of *F* and the transitivity of order along the real line, it suffices to show this in the limit case, when a=x and y=b. In this case, *L* and *R* are the tangent lines of *F* at $t=\sqrt{x}$ and $t=\sqrt{y}$, as shown in Figure 5. Let ℓ=L(1) and r=R(1) be the coordinates of the points at which *L* and *R* intersect the vertical line t=1, and note that r≤0≤ℓ by convexity of $\bar{F}$. The convexity of this function furthermore implies

(63) $$\begin{array}{cc} 1-\sqrt{x} & >\sqrt{y}-1, \end{array}$$

(64) $$\begin{array}{cc} |L^{\prime }\left(\sqrt{x}\right)| & <|R^{\prime }\left(\sqrt{y}\right)|. \end{array}$$

To compare the absolute values of *ℓ* and *r*, we express both as integrals:

(65) $$\begin{array}{cc} \ell & =\int_{t=\sqrt{x}}^{1}\left[F^{\prime }\left(\sqrt{x}\right)-F^{\prime }\left(t\right)\right]\;dt, \end{array}$$

(66) $$\begin{array}{cc} r & =\int_{t=1}^{\sqrt{y}}\left[F^{\prime }\left(t\right)-F^{\prime }\left(\sqrt{y}\right)\right]\;dt; \end{array}$$

see Figure 4 on the right. The concavity of ${\bar{F}}^{\prime }$ together with (63) implies |ℓ|≥|r|, and since *L* has smaller absolute slope than *R* (64), it follows that the two lines intersect above the zero line, as required. □

This concludes the proof in dimension one. The main obstacle to extending the proof to arbitrary dimension is the lack of decomposability (separability) of the minmax loss. Still, we decided to present the partial results and techniques, as they may help other researchers complete the proof in the future. In particular, techniques for extending results from one to arbitrary dimensions are present in the information theory literature; see for example the work on Pinsker’s inequality [23], which compares the relative entropy with another distance. This gives us hope that researchers in information theory may be well-equipped to extend the result to arbitrary dimension.

## 5. Extensions to Other Bregman Divergences

In this section, we provide a perspective for our results by extending them beyond the Shannon entropy. Specifically, we weaken the bounds while keeping them tight to generalize Theorem 1 to Bregman divergences, and we prove that the square root of the minmax divergence for the Burg entropy is a metric in $R_{+}$.

This way the inequality can be used in other applied contexts. For example, the Burg entropy (and the Itakura–Saito divergence it induces) are used in speech recognition [24].

**Burbea–Rao divergence.** The *Burbea–Rao divergence*, also called the Jensen–Bregman [25], is a straightforward generalization of the Jensen–Shannon divergence. Specifically, the underlying divergence is generalized from the relative entropy to a Bregman divergence. Letting F:Ω→R be a function of Legendre type generating a Bregman divergence and x,y∈Ω, we define

(67) $$\begin{array}{cc} {\mathrm{BR}}_{F}\left(x,y\right) & =\frac{1}{2}\left[D_{F}\left(x∥\mu \right)+D_{F}\left(y∥\mu \right)\right] \end{array}$$

(68) $$\begin{array}{cc} \; & =\frac{1}{2}\left[F\left(x\right)+F\left(y\right)-2F\left(\mu \right)\right], \end{array}$$

in which μ=(x+y)/2. Lemma 1 generalizes with a verbatim proof in which we substitute *F* for *E*. As done in [1], we also generalize the minmax divergence from the Kullback–Leibler divergence to a general underlying Bregman divergence $D_{F}$:

(69) $$\begin{array}{cc} {\mathrm{Mx}}_{F}\left(x,y\right) & =\min_{z\in \Omega }\max\left\{D_{F}\left(x∥z\right),D_{F}\left(y∥z\right)\right\}. \end{array}$$

When we compare the two, we get bounds that are considerably worse than for the special case of the Shannon entropy.

**Theorem 4** 
(Burbea–Rao divergence Comparison)**.** *Let F:Ω→R be a Legendre type function, and let x,y be points in Ω. Then $\frac{1}{2}{\mathrm{Mx}}_{F}\left(x,y\right)\leq {\mathrm{BR}}_{F}\left(x,y\right)\leq {\mathrm{Mx}}_{F}\left(x,y\right)$.*

**Proof.**  Let z∈Ω be the point that minimizes the right-hand side of (69). Using the generalization of Lemma 1 to the Burbea–Rao divergence, we get

(70) $$\begin{array}{cc} {\mathrm{BR}}_{F}\left(x,y\right) & \leq \frac{1}{2}\left[D_{F}\left(x∥z\right)+D_{F}\left(y∥z\right)\right]. \end{array}$$

Since $D_{F}\left(x∥z\right)=D_{F}\left(y∥z\right)$, the right-hand side of (70) is equal to ${\mathrm{Mx}}_{F}\left(x,y\right)$, which implies the claimed upper bound on the Burbea–Rao divergence. To prove the lower bound, we note that the larger of the two divergences to μ is at least as large as ${\mathrm{Mx}}_{F}\left(x,y\right)$. Hence, ${\mathrm{Mx}}_{F}\left(x,y\right)$ is at most the sum:

(71) $$\begin{array}{cc} {\mathrm{Mx}}_{F}\left(x,y\right) & \leq D_{F}\left(x∥\mu \right)+D_{F}\left(y∥\mu \right), \end{array}$$

and the claimed lower bound follows because the right-hand side of (71) evaluates to $2{\mathrm{BR}}_{F}\left(x,y\right)$. □

**Remark 5.** 
*The bounds in Theorem 4 are tight. To see this for the lower bound, we consider $F\left(t\right)=t+\frac{1}{t}$, which is strictly convex, differentiable, and with minimum at t=1. Setting x=ε, $y=\frac{1}{\varepsilon }$ for 0<ε<1, we have $F\left(x\right)=F\left(y\right)=\varepsilon +\frac{1}{\varepsilon }$, which implies that t=1 minimizes the maximum divergence from x and y. Some computations show that the divergences are*

(72) $$\begin{array}{cc} {\mathrm{Mx}}_{F}\left(x,y\right) & =F\left(x\right)-F\left(1\right)=\varepsilon -2+\frac{1}{\varepsilon }, \end{array}$$

(73) $$\begin{array}{cc} {\mathrm{BR}}_{F}\left(x,y\right) & =\frac{F(x)+F(y)}{2}-F\left(\mu \right)=\frac{\varepsilon^{2}+1}{2\varepsilon }-\frac{2\varepsilon }{\varepsilon^{2}+1}. \end{array}$$

*For ε→0, the ratio of ${\mathrm{BR}}_{F}\left(x,y\right)$ over ${\mathrm{Mx}}_{F}\left(x,y\right)$ goes to $\frac{1}{2}$, as required. To see the upper bound, we choose $F\left(t\right)=t^{2}$ for which ${\mathrm{BR}}_{F}\left(x,y\right)={\mathrm{Mx}}_{F}\left(x,y\right)$ for all x,y∈R.*

**Minmax divergence for Burg entropy.** Another significant entropy appearing in this context is the *Burg entropy*: B(x)=x−ln(x)+1, for x>0. Let us denote the corresponding minmax divergence my $M_{B}$.

**Theorem 5** 
(Metric for Burg Entropy)**.** *Let $F:R_{+}\to R$ be the Burg entropy. Then $\sqrt{M_{B}}$ is a metric in $R_{+}$.*

**Proof.**  The proof follows a similar structure as the proof of Theorem 3. Setting up the ratio method, we define

(74) $$\begin{array}{cc} f(x,y) & =\frac{\sqrt{M_{B}\left(x,y\right)}}{\left|\ln x-\ln y\right|}, \end{array}$$

with the denominator being the induced path metric. By Lemma 5, we have to prove that *f* is monotonic.

Next we simplify. Specifically, because $D_{B}\left(x∥y\right)=D_{B}\left(kx∥ky\right)$, we have f(kx,ky)=f(x,y) for all k>0. We may therefore assume all intervals considered by the Ratio Method to be canonical, i.e., B(x)=B(y). In such case, we have $M_{B}\left(x,y\right)=B\left(x\right)=B\left(y\right)$.

**Figure 6:**
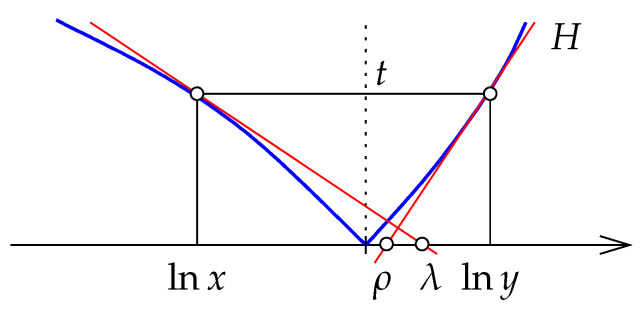
The two branches of the function *H*, two tangent lines touching the branches at points of equal height, and their intersections with the horizontal coordinate axis.

To prepare the geometric picture sketched in Figure 6, we define $H\left(x\right)=\sqrt{e^{x}-x-1}$ for x∈R. Note that f(x,y) is the aspect ratio of the rectangle in this picture. As in the proof of Theorem 3, the monotonicity of *f* is established by proving that the tangent lines to *H* at lnx and lny intersect above the horizontal axis for all canonical intervals [x,y]. To this end, it suffices to show that λ>ρ, in which

- λ is the coordinate of the intersection of the horizontal axis with the tangent on *H* at lnx;
- ρ is the coordinate of the intersection of the horizontal axis with the tangent on *H* at lny.

Reflecting the left part of *H*, we obtain an injective function $\bar{H}:R\to R$ defined by

(75) $$\begin{array}{cc} \bar{H}\left(x\right) & =\left\{\begin{array}{cc} -H(x) & \mathrm{for}\;x<0,\\ H(x) & \mathrm{for}\;0\leq x. \end{array}\right. \end{array}$$

**Figure 7:**
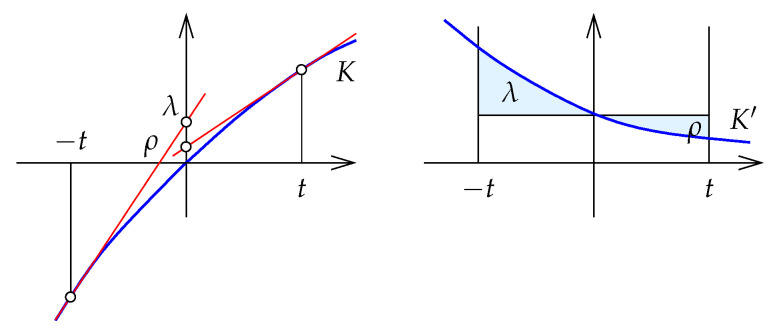
(**Left**): the graph of *K* and the intersections of the two tangent lines with the vertical coordinate axis. (**Right**): the derivative, $K^{\prime }$, and the two intersections represented as areas above and below the curve.

Note that this does not change the intersections mentioned above. Letting *K* be the inverse of $\bar{H}$, We consider the graph of *K*; see Figure 7.

Expressing this with integrals, and setting t=H(lnx)=H(lny), we can compare the two coordinates:

(76) $$\begin{array}{cc} \lambda & =K\left(-t\right)+tK^{\prime }\left(-t\right)=\int_{0}^{-t}\left[K^{\prime }\left(s\right)-K^{\prime }\left(-t\right)\right]\;ds, \end{array}$$

(77) $$\begin{array}{cc} \rho & =K\left(t\right)-tK^{\prime }\left(t\right)\;\;\;\;\;\;=\int_{0}^{t}\left[K^{\prime }\left(s\right)-K^{\prime }\left(t\right)\right]\;ds. \end{array}$$

By Lemmas A3 and A4, $K^{\prime }$ is convex and decreasing. Using the integral representation above, we interpret λ and ρ as areas; see Figure 3. It follows that λ>ρ and consequently $\sqrt{M_{B}}$ is a metric. □

## 6. Discussion

The main result of our work are the tight bounds between the minmax information divergence and the Jensen–Shannon divergence (as well as analogous results for their generalizations). The former arises naturally in the context of computational geometry and topology. Specifically, it coincides with the smallest radius of the nonempty intersection between two balls in the geometry induced by the relative entropy (which generalizes to other Bregman divergences). In this setting, this quantity is calculated repeatedly, for example to compute the one-skeleton of the Čech complex, which is one of the standard constructions in topological data analysis. In this case, our bounds are best presented as

(78) $$\begin{array}{c} \sqrt{\mathrm{JS}\left(x,y\right)}\leq \sqrt{\mathrm{Mx}\left(x,y\right)}\leq \sqrt{\frac{2}{e\ln2}\mathrm{JS}\left(x,y\right)}\approx 1.030\;\sqrt{\mathrm{JS}\left(x,y\right)}. \end{array}$$

One can therefore closely approximate the relatively costly computations of the minmax divergence, with the straightforward and efficient computations of the Jensen–Shannon divergence. Indeed, computing the minmax information requires performing a binary search or another numerical algorithm. On the other hand the formula for the Jensen–Shannon divergences is not more complex then computing the Euclidean distance.

The tightness of the bounds makes it believable that the square root of the minmax divergence may be a metric, similar to the Jensen–Shannon divergence. This turned out to be true in the one dimensional case, but the general case of *n*-dimensional spaces appears to be significantly more difficult. One key reason is that—unlike the Jensen–Shannon divergence—the minmax divergence is not decomposable (separable). We leave this case open and hope that a combinations of the proposed geometric proof techniques with additional techniques may eventually lead to a successful resolution.

## Data Availability

No new data were created or analyzed in this study. Data sharing is not applicable to this article.

## Appendix A. First Curve Discussion

In this appendix, we prove that the function $\bar{F}$ used in the proof of Theorem 3 is convex and that its derivative is concave.

**Convexity of $\bar{F}$.** Recall that the functions $F,\bar{F}:R_{+}\to R$ used in the proof of Theorem 3 are defined by

(A1) $$\begin{array}{cc} F(t) & =\sqrt{E(t^{2})+1}, \end{array}$$

(A2) $$\begin{array}{cc} \bar{F}\left(t\right) & =\left\{\begin{array}{cc} -F(t) & \mathrm{for}\;0<t\leq 1,\\ F(t) & \mathrm{for}\;1\leq t, \end{array}\right. \end{array}$$

in which E(t)=tlnt−t is the Shannon entropy; see Figure 4, left.

**Lemma A1** 
(Convexity of Function)**.** *The function $\bar{F}:R_{+}\to R$ is convex and differentiable.*

**Proof.** We aim at proving that ${\bar{F}}^{\prime \prime }\left(t\right)$ is positive for all t>0. This is equivalent to ${\bar{F}}^{\prime \prime }{\left(t\right)}^{2}=F^{\prime \prime }{\left(t\right)}^{2}$ being positive for all t>0, provided the second derivative is continuous and positive at some t>0. Indeed, for ${\bar{F}}^{\prime \prime }$ to change sign, its square must vanish. We compute

(A3) $$\begin{array}{cc} F^{\prime }\left(t\right) & =\frac{2t\ln t}{F(t)}, \end{array}$$

(A4) $$\begin{array}{cc} F^{\prime \prime }\left(t\right) & =\frac{\left(2\ln t+2\right)F\left(t\right)-2t\ln tF^{\prime }\left(t\right)}{F{\left(t\right)}^{2}} \end{array}$$

(A5) $$\begin{array}{cc} \; & =\frac{2[\left(t^{2}+1\right)\ln t-\left(t^{2}-1\right)]}{F{\left(t\right)}^{3}}, \end{array}$$

noting that $F^{\prime \prime }\left(e\right)=4/{\left(e^{2}+1\right)}^{3/2}$, which is positive as required. The numerator is twice $a\left(t\right)=\left(t^{2}+1\right)\ln t-\left(t^{2}-1\right)$, which vanishes iff $\ln t=\left(t^{2}-1\right)/\left(t^{2}+1\right)$. The derivatives on the two sides satisfy $1/t<4t/{\left(t^{2}+1\right)}^{2}$ for all positive t≠1 because ${\left(t^{2}-1\right)}^{2}>0$ for all positive t≠1. Since a(1)=0, this implies that t=1 is the only positive root of the numerator. It follows that ${F^{\prime \prime }}^{2}$ is positive everywhere, except possibly at t=1, where we get $F^{\prime \prime }\left(1\right)=\frac{0}{0}$. We settle this case with the rule de l’Hôpital, computing derivatives of the numerator and the denominator. We begin with the numerator:

(A6) $$\begin{array}{cc} a^{\prime }\left(t\right) & =2t\ln t+\frac{t^{2}+1}{t}-2t, \end{array}$$

(A7) $$\begin{array}{cc} a^{\prime \prime }\left(t\right) & =2\ln t+\frac{t^{2}-1}{t^{2}}, \end{array}$$

(A8) $$\begin{array}{cc} a^{\prime \prime \prime }\left(t\right) & =\frac{2}{t}+\frac{2}{t^{3}}, \end{array}$$

with $a\left(1\right)=a^{\prime }\left(1\right)=a^{\prime \prime }\left(1\right)=0$ and $a^{\prime \prime \prime }\left(1\right)=4$. Deriving $N=a^{2}$, we get a structure similar to the Pascal triangle with a non-zero term after six steps:

(A9) $$\begin{array}{cc} N^{\prime } & =2aa^{\prime }, \end{array}$$

(A10) $$\begin{array}{cc} N^{\prime \prime } & =2{a^{\prime }}^{2}+2aa^{\prime \prime }, \end{array}$$

(A11) $$\begin{array}{cc} N^{\prime \prime \prime } & =6a^{\prime }a^{\prime \prime }+2aa^{\prime \prime \prime }, \end{array}$$

(A12) $$\begin{array}{cc} N^{\prime \prime \prime \prime } & =6{a^{\prime \prime }}^{2}+8a^{\prime }a^{\prime \prime \prime }+2aa^{\prime \prime \prime \prime }, \end{array}$$

(A13) $$\begin{array}{cc} N^{\prime \prime \prime \prime \prime } & =20a^{\prime \prime }a^{\prime \prime \prime }+10a^{\prime }a^{\prime \prime \prime \prime }+2aa^{\prime \prime \prime \prime \prime }, \end{array}$$

(A14) $$\begin{array}{cc} N^{\prime \prime \prime \prime \prime \prime } & =20{a^{\prime \prime \prime }}^{2}+30a^{\prime \prime }a^{\prime \prime \prime \prime }+12a^{\prime }a^{\prime \prime \prime \prime \prime }+2aa^{\prime \prime \prime \prime \prime \prime }. \end{array}$$

All derivatives of *a* are well defined and take on finite values at t=1. Since $a\left(1\right)=a^{\prime }\left(1\right)=a^{\prime \prime }\left(1\right)=0$ and $a^{\prime \prime \prime }\left(1\right)\neq 0$, all derivatives of *N* vanish at t=1, except for the last, which is $N^{\prime \prime \prime \prime \prime \prime }\left(1\right)=20a^{\prime \prime \prime }{\left(t\right)}^{2}=320$. To do the same for the denominator in (A5), we set $c\left(t\right)=F{\left(t\right)}^{2}=2t^{2}\ln t-t^{2}+1$ and compute two derivatives,

(A15) $$\begin{array}{cc} c^{\prime }\left(t\right) & =4t\ln t, \end{array}$$

(A16) $$\begin{array}{cc} c^{\prime \prime }\left(t\right) & =4\ln t+4, \end{array}$$

with $c\left(1\right)=c^{\prime }\left(1\right)=0$ and $c^{\prime \prime }\left(1\right)=4$. Starting with $D=c^{3}$, we thus get

(A17) $$\begin{array}{cc} D^{\prime } & =3c^{2}c^{\prime }, \end{array}$$

(A18) $$\begin{array}{cc} D^{\prime \prime } & =6c{c^{\prime }}^{2}+3c^{2}c^{\prime \prime }, \end{array}$$

(A19) $$\begin{array}{cc} D^{\prime \prime \prime } & =6{c^{\prime }}^{3}+18cc^{\prime }c^{\prime \prime }+3c^{2}c^{\prime \prime \prime }, \end{array}$$

(A20) $$\begin{array}{cc} D^{\prime \prime \prime \prime } & =36{c^{\prime }}^{2}c^{\prime \prime }+18c{c^{\prime \prime }}^{2}+24cc^{\prime }c^{\prime \prime \prime }+3c^{2}c^{\prime \prime \prime \prime }, \end{array}$$

(A21) $$\begin{array}{cc} D^{\prime \prime \prime \prime \prime } & =90c^{\prime }{c^{\prime \prime }}^{2}+60{c^{\prime }}^{2}c^{\prime \prime \prime }+60cc^{\prime \prime }c^{\prime \prime \prime }+30cc^{\prime }c^{\prime \prime \prime \prime } \end{array}$$

(A22) $$\begin{array}{cc} \; & \;\;\;\;+3c^{2}c^{\prime \prime \prime \prime \prime }, \end{array}$$

(A23) $$\begin{array}{cc} D^{\prime \prime \prime \prime \prime \prime } & =90{c^{\prime \prime }}^{3}+360c^{\prime }c^{\prime \prime }c^{\prime \prime \prime }+90{c^{\prime }}^{2}c^{\prime \prime \prime \prime }+60c{c^{\prime \prime \prime }}^{2} \end{array}$$

(A24) $$\begin{array}{cc} \; & \;\;\;\;+90cc^{\prime \prime }c^{\prime \prime \prime \prime }+36cc^{\prime }c^{\prime \prime \prime \prime \prime }+3c^{2}c^{\prime \prime \prime \prime \prime \prime }. \end{array}$$

All derivatives of *c* are well defined and take on finite values at t=1. Since $c\left(1\right)=c^{\prime }\left(1\right)=0$ and $c^{\prime \prime }\left(1\right)\neq 0$, all derivatives of *D* vanish at t=1, except for the last, which is $D^{\prime \prime \prime \prime \prime \prime }\left(1\right)=90c^{\prime \prime }{\left(1\right)}^{3}=5,760$. We conclude that ${F^{\prime \prime }}^{2}$ is positive at t=1, namely $F^{\prime \prime }{\left(1\right)}^{2}=\frac{4\cdot 320}{5,760}=0.222\ldots$. This implies that $\bar{F}$ is convex as well as differentiable. □

**Concavity of ${\bar{F}}^{\prime }$.** Since $\bar{F}$ is differentiable, its derivative ${\bar{F}}^{\prime }:R_{+}\to R$ exists; see Figure 4, right.

**Lemma A2** 
(Concavity of Derivative)**.** *The function ${\bar{F}}^{\prime }:R_{+}\to R$ is concave.*

**Proof.** By definition, ${\bar{F}}^{\prime }$ is concave iff ${\bar{F}}^{\prime \prime }$ is monotonically decreasing, which is implied if ${\bar{F}}^{\prime \prime 2}=F^{\prime \prime 2}$ is monotonically decreasing. Recall from (A5) that $F^{\prime \prime }{\left(t\right)}^{2}=4a{\left(t\right)}^{2}/F{\left(t\right)}^{6}$. The derivative of the squared second derivative is therefore

(A25) $$\begin{array}{cc} {\left(F^{\prime \prime }{\left(t\right)}^{2}\right)}^{\prime } & =\frac{8a\left(t\right)a^{\prime }\left(t\right)F{\left(t\right)}^{6}-24a{\left(t\right)}^{2}F{\left(t\right)}^{5}F^{\prime }\left(t\right)}{F{\left(t\right)}^{12}} \end{array}$$

(A26) $$\begin{array}{cc} \; & =\frac{a(t)}{t}\cdot \frac{8F{\left(t\right)}^{4}-48a\left(t\right)t^{2}\ln t}{F{\left(t\right)}^{8}}, \end{array}$$

in which we get (A26) using $a^{\prime }\left(t\right)=F{\left(t\right)}^{2}/t$ and $F\left(t\right)F^{\prime }\left(t\right)=2t\ln t$. To continue, we write b(t) for the numerator of the second factor in (A26). Since $tF{\left(t\right)}^{8}$ is non-negative and the sign of a(t) is the same as the sign of t−1, it suffices to show that the sign of b(t) is that same as the sign of 1−t. But b(1)=0, so it is enough to show that *b* is monotonically decreasing, which it is iff $b_{1}\left(t\right)=b\left(\sqrt{t}\right)/4$ is monotonically decreasing. We get

(A27) $$\begin{array}{cc} b_{1}\left(t\right) & =2F{\left(\sqrt{t}\right)}^{4}-6a\left(\sqrt{t}\right)t\ln t\\ \; & =2{\left[t\ln t-t+1\right]}^{2} \end{array}$$

(A28) $$\begin{array}{cc} \; & \;\;\;\;-3[(t+1)\ln t-2(t-1)]t\ln t \end{array}$$

(A29) $$\begin{array}{cc} \; & =\left(2t^{2}-2t\right)\ln t-\left(t^{2}+3t\right)\ln^{2}t+2{\left(t-1\right)}^{2}, \end{array}$$

in which we use $F{\left(\sqrt{t}\right)}^{2}=E\left(t\right)+1=t\ln t-t+1$ as well as $a\left(\sqrt{t}\right)=\frac{1}{2}\left(t+1\right)\ln t-\left(t-1\right)$ to get (A28). Computing the derivative, we get

(A30) $$\begin{array}{cc} b_{1}^{\prime }\left(t\right) & =\left(2t-8\right)\ln t-\left(2t+3\right)\ln^{2}t+6t-6, \end{array}$$

which we need to be non-positive. Since $b_{1}^{\prime }\left(1\right)=0$, it suffices to show that the sign of $b_{1}^{\prime \prime }\left(t\right)$ agrees with the sign of 1−t. Computing the second derivative, we get

(A31) $$\begin{array}{cc} b_{1}^{\prime \prime }\left(t\right) & =-\frac{2}{t}\left[\left(t+3\right)\ln t+t\ln^{2}t-4t+4\right]. \end{array}$$

The first factor, $-\frac{2}{t}$, is always negative, so we just need to show that the second factor, which we write as $b_{2}\left(t\right)$, has the same sign as t−1. This is indeed the case, which we see because $b_{2}\left(1\right)=0$ and

(A32) $$\begin{array}{cc} b_{2}^{\prime }\left(t\right) & =\ln^{2}t+3\ln t-3+\frac{3}{t} \end{array}$$

(A33) $$\begin{array}{cc} \; & =\ln^{2}t+\frac{3}{t}\left[E\left(t\right)+1\right] \end{array}$$

is non-negative for all $t\in R_{+}$. We summarize by recalling the chain of implications from back to front:

- $b_{2}^{\prime }\left(t\right)$ is non-negative and $\mathrm{sign}\left(b_{2}\left(t\right)\right)=\mathrm{sign}\left(t-1\right)$,
- $\mathrm{sign}\left(b_{1}^{\prime \prime }\left(t\right)\right)=\mathrm{sign}\left(1-t\right)$ and $b_{1}^{\prime }\left(t\right)$ is non-positive,
- sign(b(t))=sign(1−t) and ${\left(F^{\prime \prime }{\left(t\right)}^{2}\right)}^{\prime }$ is non-positive,
- $F^{\prime \prime 2}$ and ${\bar{F}}^{\prime \prime }$ are monotonically decreasing,
- and finally, ${\bar{F}}^{\prime }$ is concave.

The only remaining uncertainty is at t=1, where both the numerator and the denominator of $F^{\prime \prime }$ vanishes. But as shown in the proof of Lemma A1, $F^{\prime \prime }{\left(1\right)}^{2}=0.222\ldots$ is well defined, which implies that ${\bar{F}}^{\prime }$ is differentiable at t=1 and thus concave over all of $R_{+}$. □

## Appendix B. Second Curve Discussion

Recall that *K* is the inverse function of $\bar{H}$ used in the proof of Theorem 5. In this appendix, we prove that the derivative of *K* is convex and decreasing.

**Function *K*.** Recall that the functions $H,\bar{H}:R\to R$ used in the proof of Theorem 5 are defined by

(A34) $$\begin{array}{cc} H(x) & =\sqrt{e^{x}-x-1}, \end{array}$$

(A35) $$\begin{array}{cc} \bar{H}\left(x\right) & =\left\{\begin{array}{cc} -H(x) & \mathrm{for}\;x<0,\\ H(x) & \mathrm{for}\;0\leq x. \end{array}\right. \end{array}$$

It is easy to check that $\bar{H}$ is continuously differentiable with a positive derivative. By the Inverse Function Theorem, its inverse, *K*, exists and is continuously differentiable.

**Lemma A3** 
(Convexity of Derivative)**.** *The derivative of the function K:R→R is convex.*

**Proof.** Consider first the case x>0, introduce $y\left(x\right)=\sqrt{e^{x}-x-1}$, and note that $y^{\prime }\left(x\right)=\frac{\;dy}{\;dx}=\frac{e^{x}-1}{2y(x)}$. Setting $x^{\prime }\left(y\right)=\frac{\;dx}{\;dy}=\frac{2y}{e^{x}-1}$, we prove that $x^{\prime }\left(y\right)$ is convex or, equivalently, that $x^{\prime \prime }\left(y\right)$ is increasing. Using the definition of y(x), we get

(A36) $$\begin{array}{cc} x^{\prime \prime }\left(y\left(x\right)\right) & =2\frac{\left(e^{x}-1\right)-ye^{x}x^{\prime }\left(y\right)}{{\left(e^{x}-1\right)}^{2}} \end{array}$$

(A37) $$\begin{array}{cc} \; & =2\frac{-e^{2x}+2xe^{x}+1}{{\left(e^{x}-1\right)}^{3}}. \end{array}$$

Since both x(y) and y(x) are increasing functions, it suffices to show that $g_{1}\left(x\right)=x^{\prime \prime }\left(y\left(x\right)\right)$ is increasing. Note that

(A38) $$\begin{array}{cc} g_{1}^{\prime }\left(x\right) & =\frac{e^{x}}{{\left(e^{x}-1\right)}^{4}}\;g_{2}\left(x\right), \end{array}$$

in which $g_{2}\left(x\right)=e^{x}-4e^{x}\left(x-1\right)-2x-5$. At this point, we need to prove that $g_{2}\left(x\right)$ is positive. Observe that $g_{2}^{\prime \prime }\left(x\right)=4e^{x}\left(e^{x}-x-1\right)>0$ and $g_{2}\left(0\right)=g_{2}^{\prime }\left(0\right)=0$, thus $g_{2}\left(x\right)>0$ for x>0. Tracing back the chain of derivations, we conclude that $K^{\prime }=x^{\prime }\left(y\right)$ is convex. Consider second the case x<0, introduce $y\left(x\right)=-\sqrt{e^{x}-x-1}$, and note that $y^{\prime }\left(x\right)=\frac{\;dy}{\;dx}=\frac{e^{x}-1}{2y(x)}$, as above. Hence, we can repeat the steps using the same functions but now for x<0. We conclude that $x^{\prime }\left(y\right)$ is convex for x>0 and for x<0. We know that it is continuous everywhere, and continuously differentiable for x≠0 by the calculations above. Using (A36) and (A37), we may also verify that $x^{\prime \prime }\left(y\right)$ is continuous at y=0, hence everywhere. This implies that $K=x^{\prime }\left(y\right)$ is convex on R. □

**Lemma A4** 
(Decreasing Derivative)**.** *The derivative of the function K:R→R is decreasing.*

**Proof.** Consider first x>0. By (A36) and (A37), it suffices to prove that

(A39) $$\begin{array}{cc} g_{3}\left(x\right) & =-e^{2x}+2xe^{x}+1<0. \end{array}$$

Indeed, this holds as $g_{3}^{\prime }\left(x\right)=-2e^{x}\left(e^{x}-\left(x+1\right)\right)<0$ and $g_{3}^{\prime }\left(0\right)=0$. Since the derivative of *K* is convex, for all *x*, and decreasing, for x>0, it must also be decreasing for x≤0. □
